# Improving Employee Well-Being and Effectiveness: Systematic Review and Meta-Analysis of Web-Based Psychological Interventions Delivered in the Workplace

**DOI:** 10.2196/jmir.7583

**Published:** 2017-07-26

**Authors:** Stephany Carolan, Peter R Harris, Kate Cavanagh

**Affiliations:** ^1^ School of Psychology University of Sussex Brighton United Kingdom

**Keywords:** adherence, engagement, Internet, meta-analysis, psychological interventions, stress, systematic review, wellbeing, workplace

## Abstract

**Background:**

Stress, depression, and anxiety among working populations can result in reduced work performance and increased absenteeism. Although there is evidence that these common mental health problems are preventable and treatable in the workplace, uptake of psychological treatments among the working population is low. One way to address this may be the delivery of occupational digital mental health interventions. While there is convincing evidence for delivering digital psychological interventions within a health and community context, there is no systematic review or meta-analysis of these interventions in an occupational setting.

**Objective:**

The aim of this study was to identify the effectiveness of occupational digital mental health interventions in enhancing employee psychological well-being and increasing work effectiveness and to identify intervention features associated with the highest rates of engagement and adherence.

**Methods:**

A systematic review of the literature was conducted using Cochrane guidelines. Papers published from January 2000 to May 2016 were searched in the PsychINFO, MEDLINE, PubMed, Science Direct, and the Cochrane databases, as well as the databases of the researchers and relevant websites. Unpublished data was sought using the Conference Proceedings Citation Index and the Clinical Trials and International Standard Randomized Controlled Trial Number (ISRCTN) research registers. A meta-analysis was conducted by applying a random-effects model to assess the pooled effect size for psychological well-being and the work effectiveness outcomes. A positive deviance approach was used to identify those intervention features associated with the highest rates of engagement and adherence.

**Results:**

In total, 21 randomized controlled trials (RCTs) met the search criteria. Occupational digital mental health interventions had a statistically significant effect post intervention on both psychological well-being (*g*=0.37, 95% CI 0.23-0.50) and work effectiveness (*g*=0.25, 95% CI 0.09-0.41) compared with the control condition. No statistically significant differences were found on either outcome between studies using cognitive behavioral therapy (CBT) approaches (as defined by the authors) compared with other psychological approaches, offering guidance compared with self-guidance, or recruiting from a targeted workplace population compared with a universal workplace population. In-depth analysis of the interventions identified by the positive deviance approach suggests that interventions that offer guidance are delivered over a shorter time frame (6 to 7 weeks), utilize secondary modalities for delivering the interventions and engaging users (ie, emails and text messages [short message service, SMS]), and use elements of persuasive technology (ie, self-monitoring and tailoring), which may achieve greater engagement and adherence.

**Conclusions:**

This review provides evidence that occupational digital mental health interventions can improve workers’ psychological well-being and increase work effectiveness. It identifies intervention characteristics that may increase engagement. Recommendations are made for future research, practice, and intervention development.

## Introduction

### Background

Nearly one in three workers in Europe [1] and the United States [2] report that they are affected by stress at work. Work-related stress, depression, and anxiety can result in reduced work performance and absenteeism [3-7], costing an estimated 3% to 4% of gross national product [1]. There is evidence that these conditions are both preventable and treatable in the workplace [8-9] and that workers who receive treatment are more likely to be highly productive [10,11].

The workplace has been identified as a potentially ideal site for delivering mental health prevention programs [12] and increasing access to appropriate treatment [7], resulting in a benefit to both employees and employers [11]. However, uptake of psychological treatments among the working population is low [10], with one study reporting that only 15% of workers with a mental health problem had sought help in the preceding month [13], resulting in many depressed workers going untreated or being inadequately treated [11]. Help seeking among the working population has been reported at between 43% [10] and 15% [13]. People are increasingly turning to the Internet for health care information [14], prevention, and treatment [15]. Although there is convincing empirical evidence for the effectiveness of evidence-based digital psychological interventions delivered within a health and community context, the evidence for digital interventions delivered in a workplace setting is less clear [16].

Several meta-analyses and systematic reviews have found evidence for the effectiveness of digital psychological interventions delivered in nonworkplace settings for common mental health problems including depression, anxiety [17-23], and stress in adults [24], but these reviews do not focus on the delivery of these interventions to working adults or in the workplace. We suggest that the delivery of occupational health interventions is different to the delivery of interventions in health or community settings and that the context of the workplace is likely to impact on the way that these interventions are delivered and received, and is therefore, likely to impact on their effectiveness. To our knowledge no previous systematic review has specifically reported on digital interventions for stress and mental health in the workplace.

This systematic review and meta-analysis seeks to address this gap in the literature by identifying studies that deliver digital occupational mental health interventions and evaluating their effectiveness at increasing employee psychological well-being (by targeting a reduction in stress, depression, and psychological distress) and work effectiveness.

Engagement and adherence are two of the major challenges to delivering and evaluating Web-based interventions [25-27]. Boosting engagement and adherence with Web-based interventions increases the extent to which users are exposed to the content and may be an important determinant of effectiveness [28] and a consistent predictor of positive outcomes [29-31].

This review uses a positive deviance approach (eg, [32,33]) to identify the intervention features that are associated with the highest levels of intervention engagement and adherence in the workplace context.

### Aims of This Review

The aims of this systematic review and meta-analysis are to evaluate the overall effectiveness of occupational digital mental health interventions for employee psychological well-being and work effectiveness and to identify, through the partial implementation of positive deviance methodology, which intervention features influence engagement and adherence. To this end, the review will address the following three questions:

1. Are occupational digital mental health interventions associated with lower levels of stress and mental health symptoms post intervention than control groups?
2. Are occupational digital mental health interventions associated with increased work effectiveness post intervention?
3. Which intervention features are associated with the highest levels of engagement and adherence?

## Methods

### Search Strategy

This review was conducted following the Cochrane guidance for systematic reviews [34]. We searched PsychINFO, MEDLINE, PubMed, Science Direct, and the Cochrane database of systematic reviews for relevant studies published from January 2000 to May 2016. The key terms used for these searches are displayed in Table 1. To increase coverage, we searched the databases of the researchers, relevant websites (eg, the Health and Safety Executive, the Faculty of Occupational Medicine, and the National Institute for Heath and Care Excellence), reference lists of included studies, and relevant journals. Unpublished data was sought using the Conference Proceedings Citation Index and the Clinical Trials and ISRCTN research registers. Three potentially relevant trials were identified through the research registers, and the researchers were contacted. However, no additional data from these unpublished studies became available.

**Table 1:** Search terms.

(stress OR	AND (intervention OR	AND (online OR	AND (Workplace OR
resilien* “mental health” depress* anxiety “mental illness” burnout “psychological ill health” “mental disorder” “mood disorder”)	“stress management” “stress inoculation training” resilience “problem solving” self-help CBT “cognitive behav* therapy”)	Internet web-based app computer)	“work place” “occupational health” worker* employee* business* staff work “work related” job*)

### Inclusion and Exclusion Criteria

To meet the aims of this review, a study had to meet the following criteria: (1) use a randomized controlled design; (2) utilize a nontreatment, treatment as usual, or active control; (3) aimed at employed participants aged 18 years or over; (4) comprise a psychological intervention aimed at increasing psychological well-being (eg, by reducing symptoms of stress or depression) or work effectiveness (eg, by increasing engagement or productivity); (5) be delivered via the Internet, mobile technology, or a computer program; (6) written in English; and (7) offer sufficient post intervention data (sample sizes, means, and standard deviations [SDs] for both the control and the treatment condition) in the paper or by contacting the authors to calculate the effect size for either a well-being or a work effectiveness outcome.

Studies were excluded if they exclusively targeted people on extended sick leave or were targeting populations with complex mental health problems including post-traumatic stress disorder (PTSD), schizophrenia, or comorbid substance misuse. Studies were also excluded if technology was used purely as a medium for communication (eg, Skype, videoconference, e-counseling): the active element of the intervention had to be delivered on the Web or via mobile technology. Studies were also excluded if homework was completed on the Web but the intervention was delivered in person.

### Data Extraction

The data was coded at four levels: study, intervention, participants, and outcomes. Further information about coding categories is available from the study registration (the protocol for this systematic review and meta-analysis was registered with the International Prospective Register of Systematic Reviews (PROSPERO; registration number CRD42016033935).

### Data Analysis

The number of participants and the between group, post intervention means, and SD for the control and the experimental group on selected psychological measures (measures prioritized in the order: stress, depression, and psychological distress) and selected work effectiveness measures (prioritized in the order: work engagement, productivity or job specific effectiveness, work related self-efficacy, and work related rumination) were entered into Review Manager (RevMan) version 5.3 and SPSS version 22 (IBM Corp). Where more than one measure was available, the measures were prioritized in the order given above. Forest plots of the between group, post intervention effect size (Hedges *g*) for both outcome variables (psychological well-being and work effectiveness) were produced using RevMan. The magnitude of effect size was interpreted using the classification given by Cohen (small=0.2, medium=0.5, and large=0.8) [35].

To test for the presence of heterogeneity of effect size, we used the chi-square (χ^2^) and the heterogeneity (I^2^) statistics. A large χ^2^ relative to its degree of freedom and a low *P* value provides evidence of heterogeneity [34]. An I^2^ value of 25% suggests that heterogeneity is low, 50% suggests medium, and 75% suggests high [36]. Since we expected considerable heterogeneity, a random effects model was performed [37]. Heterogeneity was explored using subgroup analyses. Possible moderating factors included (1) therapeutic approach (cognitive behavioral therapy [CBT] vs other), (2) guidance (guided vs nonguided), and (3) population (targeted vs universal). Interventions were coded as using CBT if the authors of the studies described the therapeutic approach as cognitive or cognitive behavior and as guided if guidance from a person was described. We coded the population as targeted if the inclusion criteria included elevated levels of stress, depression, or insomnia. Publication bias was tested using funnel plots for both outcome measures.

### Risk of Bias Assessment

An assessment of the methodological quality of the studies included in this review was conducted using the Cochrane Collaboration’s risk of bias tool [34]. The tool assesses possible sources of bias using seven main categories: (1) random sequence generation, (2) allocation concealment, (3) blinding of participants and personnel, (4) blinding of outcome assessment, (5) incomplete outcome data, (6) selective reporting, and (7) other bias. Twenty-five percent of studies were assessed by the first and second author independently, with a high rate of agreement; differences were discussed and resolved. The first author completed all subsequent bias assessments. Publication bias was assessed by appraising funnel plots for asymmetry.

### Positive Deviance

A partial implementation of the positive deviance approach was used to identify intervention features associated with the highest levels of engagement and adherence. Positive deviance is as an assets-based approach used to identify sustainable solutions to difficult problems by identifying “uncommon, beneficial practices” [33]. Bradley et al [32] describe four steps to using the positive deviance approach: (1) identify “positive deviants,” that is, organizations that consistently demonstrate exceptionally high performance in the area of interest, (2) study the organizations in depth to generate hypotheses about practices that enable organizations to achieve high performance, (3) test hypotheses with other organizations, and (4) work with other organizations to disseminate the evidence about high performance. In this study, the first two steps were adapted and applied to study interventions showing the highest levels of engagement (cf. [38]). To assess engagement, we ranked the 21 studies in this review in percentile order in terms of intervention completion and intervention group study attrition. Completion of the intervention and intervention group study attrition were seen as the most relevant and widely report measures of intervention engagement and adherence. Studies at the 70th percentile and above were selected and their interventions were reviewed in depth to generate hypotheses about intervention features that may enable high levels of engagement (hypotheses generation). This is a modification from our protocol.

## Results

### Search Results

**Figure 1:**
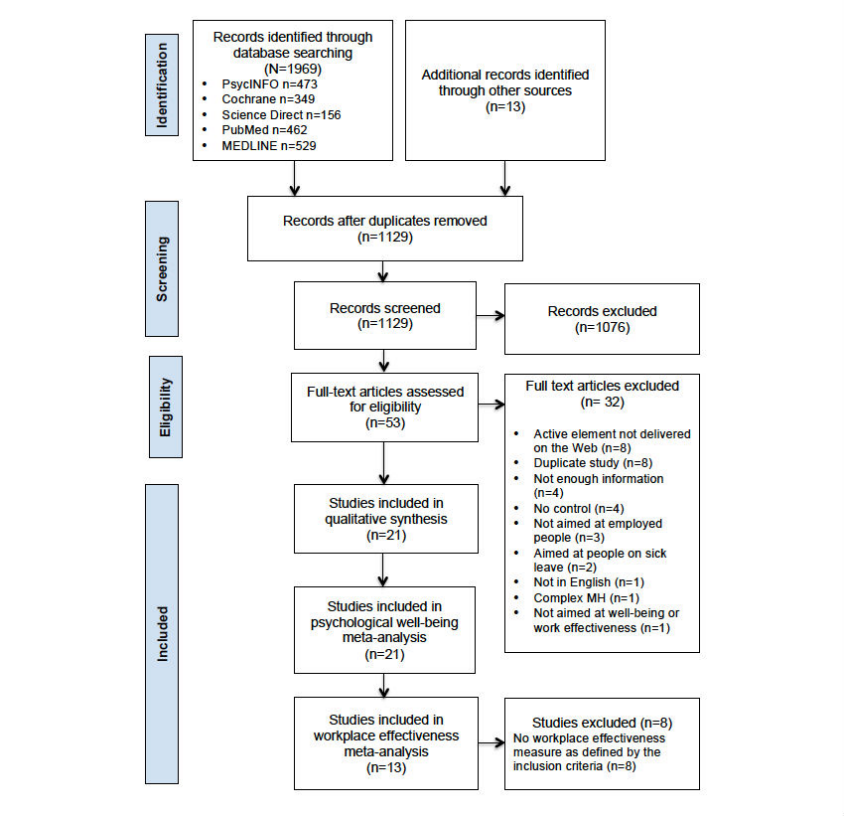
Flow diagram of study selection.

The initial search resulted in 1129 citations after duplicates had been removed. These citations were screened using the exclusion and inclusion criteria and 1076 excluded. Full papers were retrieved and examined for eligibility for the remaining 53 studies. We included 21 studies in the review: 21 in the qualitative synthesis, 21 in the psychological well-being meta-analysis, and 13 of the 21 in the work effectiveness meta-analysis. See Figure 1 for the Preferred Reporting Items for Systematic Reviews and Meta-Analyses (PRISMA) flowchart of study selection. One study [39] did not exclude unemployed participants, but the aim of the study was to assess effectiveness of cognitive behavioral treatment for work related stress; 80% of the participants were in full time work and a number were unemployed because of work-related stress or were experiencing stress in unpaid jobs. For these reasons we included the study in the review. A sensitivity analysis indicated no difference in our overall results if this study was excluded. A second study [40] examined the effects on job stress of Web-based career identity training on Japanese hospital nurses. This study was excluded from the review as it was felt that the intervention was closer to a career counseling intervention than a psychological intervention.

### Designs of the Included Studies

The 21 RCTs included in this review compared a Web-based psychological intervention delivered in the workplace with a wait list control (WLC) (71%, 15/21), an active control (19%, 4/21), or care as usual (9%, 2/21). Additionally, 17 (81%, 17/21) of the studies completed an intention-to-treat analysis, and 4 (19%, 4/21) completed a per-protocol analysis. Multimedia Appendix 1 describes the selected characteristics for the 21 identified studies.

### Risk of Bias

**Figure 2:**
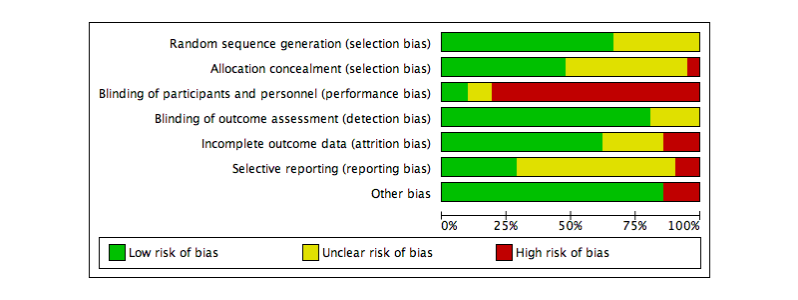
Estimated risk of bias across all studies.

Figure 2 shows an estimation of the risk of bias across all studies. Of the 21 studies included in this review, only 8 (38%) were able to fulfill 5 or more low risk of bias ratings across the seven categories used. Only 2 of the studies (9%, 2/21) were able to blind both participants and personnel to the condition allocation (performance bias), and only 6 (29%, 6/21) demonstrated low reporting bias by preregistering or making their study protocol available and by reporting all the primary outcomes. Less than half of all ratings (45.6%, 67/147) were unclear or high risk.

### Publication Bias

**Figure 3:**
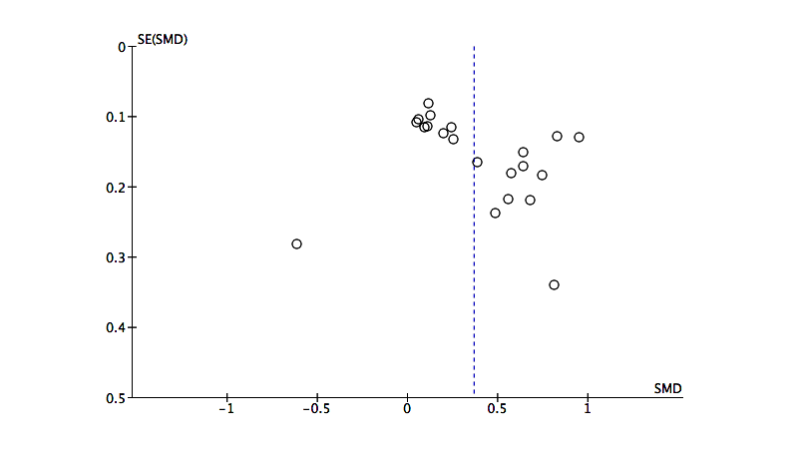
Funnel plot of post intervention effect sizes by standard error for the psychological wellbeing outcome.

**Figure 4:**
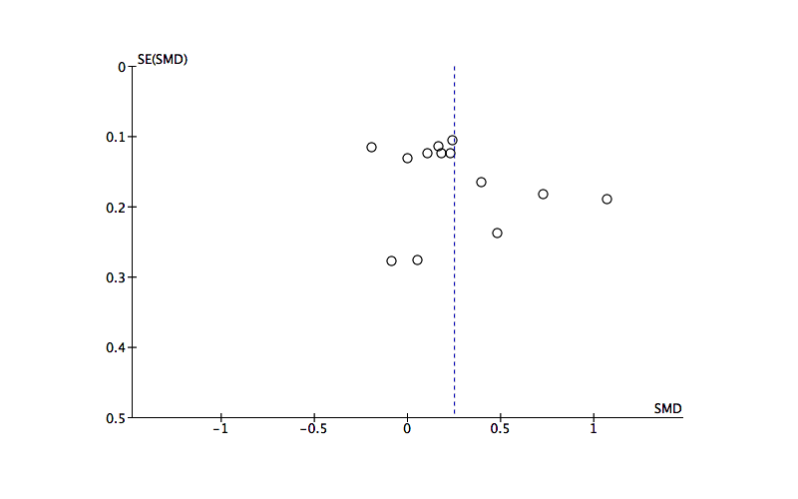
Funnel plot of post intervention effect sizes by standard error for the work effectiveness outcome.

Funnel plots for the effect sizes for the psychological wellbeing outcome and the work effectiveness outcome are shown in Figures 3 and 4, respectively. There is no indication of problematic clustering in these plots, which are fairly evenly distributed around the mean effect size, suggesting little evidence of publication bias.

### Sample and Study Characteristics

The 21 studies included in this review originated from 7 countries: 6 from the United States [41-46], 6 from Germany [47-52], 3 from the Netherlands [39,53,54], two each from the United Kingdom [55,56] and Japan [57,58], and one each from Australia [59] and Sweden [60]. Four of the studies recruited from the general working population [39,41,47,48], whereas the other studies recruited from organizations working in education [49-51], health, or local authorities [53,55,56]; a call center [42]; manufacturing [57]; technology [43,58]; sales [59]; chemicals [44]; human resource (HR) [45]; insurance [52]; and transport and communication [56]. One study recruited from organizations working in banking, research, education, and security [54]; 1 study recruited middle managers from medium and large companies [60], and another recruited employed care-givers of people with dementia [46].

The sample size in the studies ranged from 48 to 762. Overall, the studies recruited and randomized 5260 participants: 2711 to a psychological intervention delivered predominantly on the Web and 2549 to a control condition. The data for 2438 participants was analyzed in the experimental group and 2360 in the control group. The discrepancy in numbers between randomized and analyzed is accounted for by study attrition [55,56,58].

Women made up 58% (3051/5260) of all randomized participants. All the studies were aimed at a working age population. The range of mean ages reported across the studies was 36.4 to 48.4 years in the intervention groups and 34.3 to 47.8 years in the control groups. Nine of the studies (43%) recruited from a targeted population, including individuals with elevated levels of depression [41,54,56], stress [47-49], and insomnia [50,51]; one study recruited participants who had taken 10 or more consecutive days off work for stress, anxiety, or depression [55]. The remaining 12 studies (57%, 12/21) targeted a universal population with no set psychological inclusion criteria.

### Intervention Characteristics

Multimedia Appendix 2 describes the selected characteristics of the interventions used in the studies included in this review. Over half of the interventions were based on cognitive or cognitive behaviour therapy (12/21, 57%) [39,41,43,50,51, 53-59], with 3 based on stress and coping (14%) [46-48], 2 on mindfulness (10%) [42,44], and one each (5%) on social cognitive theory [45], problem solving training [49], positive psychology [52], and acceptance and commitment therapy [60]. The mean duration of the interventions was 7.6 weeks (SD=2.5; range 4.3 to 13.0). Seventeen (81%) of the interventions included in the studies used a website as their primary means of delivering the intervention [42-54,56-59], 2 (10%) delivered the intervention via a computer application [41,60], 1 (5%) via email [39], and one (5%) through a standalone computer [55]. Secondary modalities used by the studies to deliver the intervention and to engage users were email (12/21, 57%) [41,42,44,46,48,51-54,57-59], texting (4/21, 19%) [44,47,48,60], conference calls (2/21, 10%) [44,59], telephone calls (1/21, 5%) [59], face-to-face delivery (1/21, 5%) [44], a workbook (1/21, 5%) [44], and a compact disc (CD; 1/21, 5%) [42]. Just over half of the interventions (11/21, 52%) were self-guided [41-43,45-47,50,52,53,55,56], and 10 (48%, 10/21) offered users of the intervention some form of guidance: seven of those 10 studies (70%) described the guidance as coming from a therapist or coach [39,44,48,49,51,54,60], 2 (20%) were described as a coordinator or member of staff [57,59], and one (10%) as a clinical psychologist [58].

### Study Attrition and Intervention Completion

Study attrition for the control and the intervention groups separately was available for 20 of the studies (one study reported combined study attrition [46]). The mean attrition for the intervention groups was 23% (SD=16.1, range 3% to 54%) and for the control groups 13% (SD=11.6, range 0% to 41%).

Intervention completion (adherence) data was available for 19 of the studies (data not available for 2 of the studies [41,52]). Most studies reported the percentage of participants that completed all or part of the intervention. The mean adherence (taken as the highest level of completion reported by the authors) was 45% (SD=29.3, range 3% to 95%).

### Persuasive Technology

Studies were coded to see what if any elements of persuasive technology the interventions used to help support users to benefit from the intervention. They were coded using the classifications given by Fogg [61]. These are (1) reduction (reducing complex behavior to simple tasks), (2) tunneling (leading users through a predetermined sequence of actions or events), (3) tailoring (providing information relevant to specific individuals), (4) suggestion (making a suggestion at the most appropriate time), (5) self-monitoring (enabling people to monitor themselves), (6) surveillance (the use of computer technology to allow one party to monitor the behavior of another), and (7) conditioning (using technology to reinforce target behaviors). Seventeen of the 21 studies (81%) reported using a form of persuasive technology [39,41,43,44,46-51,53-55,57-60]. Tailoring was used by 57% (12/21) of interventions [39,43,44,46-51,53,54,58], self-monitoring by 43% (9/21) [41,44,47,50,51,55,57,59,60], and tunneling by 14% (3/21) [41,54,55]. We were unable to identify any forms of persuasive technology in the descriptions of 19% (4/21) of studies [42,45,52,56].

### Meta-Analyses Findings

Post intervention means, SDs, and group numbers were extracted from the 21 studies included in this review. Two separate meta-analyses were completed for (1) psychological well-being, and (2) work effectiveness. Of the 21 studies included in the psychological well-being meta-analysis, 13 were also included in the work effectiveness meta-analysis. Both analyses were conducted using a random-effects model.

**Figure 5:**
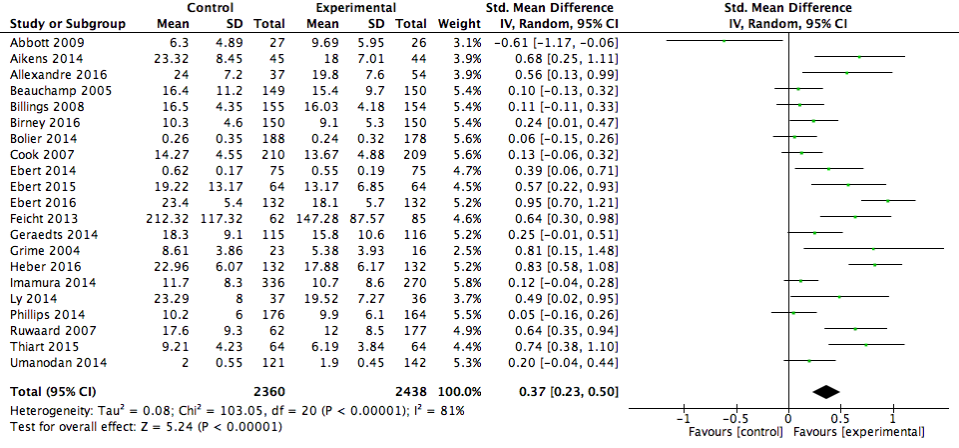
Forest plot of post intervention effect size for the psychological wellbeing outcome.

Figure 5 is a forest plot for the 21 studies that included a measure of psychological well-being. The Web-based psychological intervention delivered in the workplace resulted in significantly reduced levels of stress, depression, and psychological distress scores post intervention for the intervention condition compared with the control condition (*z*_20_=5.24, *P*<.001) with a small effect size (*g*=0.37, 95% CI 0.23-0.50). The resulting effect sizes were significantly and highly heterogeneous (χ^2^_20_=103.1 *P*<.001; I^2^=81%).

**Figure 6:**
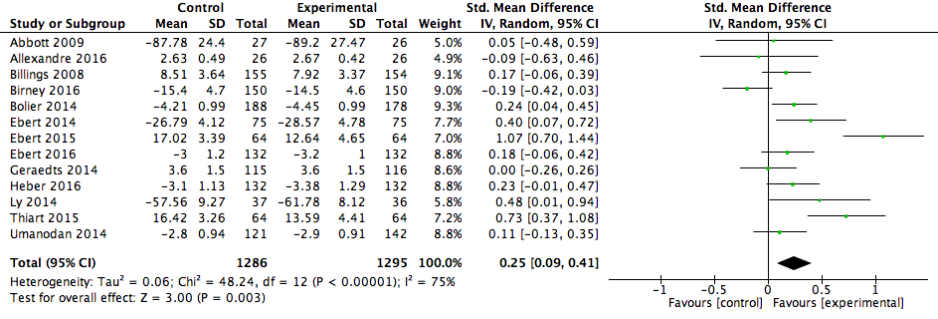
Forest plot of post intervention effect size for the work effectiveness outcome.

Figure 6 shows a forest plot for the 13 studies that included a work effectiveness measure. Participants in the intervention group showed significantly greater workplace effectiveness scores compared with those in the control conditions (*z*_12_=3.00, *P*=.003) with a small effect size (*g*=0.25, 95% CI 0.09-0.41). The resulting effect sizes were significantly and highly heterogeneous (χ^2^_12_=48.2, *P*<.001, I^2^= 75%).

The results of both meta-analyses suggested that further subgroup analyses were warranted.

### Sensitivity Analysis

We conducted outlier analysis by examining the forest plots of standard mean difference effect sizes and CIs for both the psychological well-being measure and the work effectiveness measure. One study was identified as a possible outlier on the psychological well-being outcome [59] because of its negative effect size (contrary to the other studies) and because its CIs did not fall into the range of the other studies. A sensitivity analysis excluding the study from analysis shows that the result of the main effect remains robust. A sensitivity analysis was also conducted comparing studies with lower and higher risk of bias. Studies with a low risk of bias produced larger effect sizes on the psychological well-being outcome (d=0.57, 95% CI 0.35-0.78) than studies with a high risk of bias (d=0.23, 95% CI 0.10-0.36). The groups were significantly different from each other (χ^2^_1_=6.9, *P*=.009). No significant difference was found between the two groups on the work effectiveness outcome.

### Subgroup Analyses

Subgroup analyses for both the psychological well-being and work effectiveness outcomes were conducted looking at (1) therapeutic approach (CBT vs other), (2) guidance (guided vs nonguided), and (3) population (targeted vs universal). Table 2 shows the results of the subgroup analyses.

#### Therapeutic Approach

Subgroup analysis of the psychological well-being outcome comparing studies using CBT (k=12; as described by the authors) with studies using other psychological approaches (k=9) showed that the groups were not significantly different from each other (χ^2^_1_=3.63, *P*=.06), suggesting that for the psychological well-being outcome, the psychological approach used was not a source of heterogeneity. The pooled effect size for studies using the psychological approach of CBT was small (*g*=0.25, 95% CI 0.10-0.40), and for studies using other psychological approaches it was medium (*g*=0.52, 95% CI 0.28-0.76). Both are significant effect sizes (*z*_11_=3.35, *P* ≤.001; and z_8_=4.28, *P* ≤.001, respectively).

Subgroup analysis of the work effectiveness outcome comparing studies using predominantly CBT (k=8) with studies using other psychological approaches (k=5) showed that the groups were not significantly different from each other (χ^2^_1_=0.01, *P*=.94), suggesting that for the work effectiveness outcome, the therapeutic approach was not a source of heterogeneity. The small pooled effect size for studies using CBT (*g*=0.26, 95% CI 0.01-0.50) and other psychological approaches (*g*=0.25, 95% CI 0.11-0.39) are significant (*z*_7_=2.05, *P*=.04; and z_4_=3.47, *P*≤.001, respectively).

#### Guidance

Subgroup analysis of the psychological well-being outcome comparing interventions providing guidance (k=10) with interventions that were self-guided (k=11) showed that the groups were not significantly different from each other (χ^2^_1_=0.11, *P*=.74), suggesting that for the psychological well-being outcome, guidance was not a source of heterogeneity. The pooled effect size for both groups was small (guided interventions: *g*=0.39, 95% CI 0.18-0.61; and self-guided interventions: *g*=0.34, 95% CI 0.16-0.53) both were significant effect sizes (*z*_9_=3.58, *P*≤.001; and z_10_=3.63, *P*≤.001, respectively).

Subgroup analysis of the work effectiveness outcome comparing interventions providing guidance (k=7) with interventions that were self-guided (k=6) showed that the groups were not significantly different from each other suggesting that for the work effectiveness measure, guidance was not a source of heterogeneity (χ^2^_1_=0.1, *P*=.81). The pooled effect size for studies using interventions that are guided was a small significant effect size (*g*=0.27, 95% CI 0.08-0.45; z_6_=2.84, *P*=.005). The pooled effect size for interventions that are self-guided was a small nonsignificant effect size (*g*=0.23, 95% CI −0.06 to 0.51; z_5_=1.55, *P*=.12).

**Table 2:** Results of subgroup analyses investigating the effect of therapeutic approach, guidance, and population on outcome.

Outcome		Moderator	k (n)	Intervention effects						Heterogeneity within each subgroup								
				*g*		95% CI	Z	*P*		χ^2^		df		*P*				I^2^
**Therapeutic approach**																			
	Well-being	CBT	12 (3002)	0.25		0.10-0.40	3.35	<.001		38.9		11		<.001				72%
		Other	9 (1796)	0.52		0.28-0.76	4.28	<.001		46.6		8		<.001				83%
		Test for subgroup difference							3.6		1		.06				72%	
	Work effectiveness	CBT	8 (1778)	0.26		0.01-0.50	2.05	.04		44.1		7		<.001				84%
		Other	5 (803)	0.25		0.11-0.39	3.47	<.001		3.5		4		.47				0%
		Test for subgroup difference							0.01		1		.94				0%	
**Guidance**																			
	Well-being	Guided	10 (2096)	0.39	0.18-0.61		3.58	<.001		46.6		9		<.001				81%
		Self-guided	11 (2702)	0.34	0.16-0.53		3.63	<.001		53.9		10		<.001				81%
		Test for subgroup difference							0.1		1		.74				0%	
	Work effectiveness	Guided	7 (1162)	0.27		0.08-0.45	2.84	.005		13.9		6		.03				57%
		Self-guided	6 (1419)	0.23		−0.06 to 0.51	1.55	.12		33.8		5		<.001				85%
		Test for subgroup differences							0.1		1		.81				0%	
**Population**																			
	Well-being	Targeted	9 (1844)	0.52		0.28-0.75	4.32	<.001		46.9		8		<.001				83%
		Universal	12 (2954)	0.25		0.11-0.40	3.39	<.001		37.3		11		<.001				71%
		Test for subgroup difference							3.6		1		.06				72%	
	Work effectiveness	Targeted	7 (1465)	0.32		0.04-0.61	2.21	.03		44.6		6		<.001				87%
		Universal	6 (1116)	0.18		0.06-0.30	3.00	.003		3.4		5		.64				0%
		Test for subgroup difference							0.8		1		.37				0%	

#### Population

Subgroup analysis of the psychological well-being outcome comparing a targeted working population (k=9) with a universal working population (k=12) showed that the groups were not significantly different from each other (χ^2^_1_= 3.59, *P*=.06), suggesting that for the psychological well-being outcome, population was not a source of heterogeneity. The pooled effect size for the targeted working population was medium (*g*=0.52, 95% CI 0.28-0.75) and for the universal working population it was small (*g*=0.25, 95% CI 0.11-0.40). Both were significant effect sizes (*z*_8_=4.32, *P* ≤.001 and z_11_=3.39, *P* ≤.001, respectively).

Subgroup analysis of the work effectiveness outcome comparing a targeted working population (k=7) with a universal working population (k=6) showed that the groups were not significantly different from each other (χ^2^_1_=0.81, *P*=.37), suggesting that for the work effectiveness measure, population was not a source of heterogeneity. The pooled effect size for both groups was small (targeted working population: *g*=0.32, 95% CI 0.04-0.61, and universal working population: *g*=0.18, 95% CI 0.06-0.30); both effect sizes were significant (*z*_6_=2.21, *P*=.03, and z_5_=3.00, *P*=.003, respectively).

### Positive Deviance Analysis

Of the 21 studies included in this review, 6 studies were in the 70th percentile and above for the lowest attrition in the intervention group [41,47,48,51,57,60], and 4 studies were in the 70th percentile and above for the highest intervention completion [48,49,51,57]. Three studies appeared in both groups [48,51,57], leaving 7 unique studies [41,47-49,51,57,60] that we reviewed in depth to generate hypotheses about intervention features associated with the highest levels of engagement.

The mean percentage of intervention group attrition in the high engagement group was 8% (SD 4.4), and for the other studies it was 31% (SD 14.5). The mean of the highest intervention completion reported by the authors for the high engagement group was 68% (SD 22.0) and for the other studies it was 33% (SD 26.0).

Interventions presented in the 7 studies in the high engagement group were reviewed. The interventions for 5 out of the 7 studies offered guidance (71%), compared with only 5/14 of the remaining studies (36%). The mean number of weeks that the intervention was delivered in the high engagement group was 6.6 (SD=0.54, range 6-7 weeks), compared with a mean of 8.1 (SD=3.0, range 4.3-13.0 weeks) in the other studies. All 7 of the studies in the high engagement group described the use of persuasive technology (5/7, 71% self-monitoring, 4/7, 57% tailoring, 1/7, 14% tunneling), compared with 10/14 (71%) in the remaining studies (8/14, 57% tailoring, 5/14, 29% self-monitoring, and 2/14, 14% tunneling).

Six of the 7 studies (86%) in the high engagement group utilized a secondary modality for delivering the intervention and engaging users (4 studies used emailing and 3 studies used texting), compared with only 8 of the remaining 14 studies (57%). Only 2 of the 21 studies included in this review used a mobile phone app as their primary modality for delivering the intervention; both studies were included in the high engagement group.

### Hypotheses Generation

These findings suggest that interventions that achieve the greatest engagement and adherence offer guidance, are delivered over a shorter time frame (6 to 7 weeks), utilize secondary modalities for delivering the intervention and engaging users (ie, email and text messages), and use persuasive technology (ie, self-monitoring and tailoring). There is also a suggestion that a mobile phone app is a promising modality for engaging users of occupational digital mental health interventions.

## Discussion

This review is the first meta-analysis that brings together RCTs of occupational digital mental health interventions and allows us to draw conclusions about both psychological well-being and work effectiveness outcomes. The adaptation of the positive deviance approach was helpful in enabling us to identify and explore in depth the features of high performing interventions in order to generate hypotheses about the intervention features that may promote engagement.

### Study Characteristics

The 21 studies included in this review recruited and randomized 5260 participants. They were predominantly recruited from the knowledge sector (ie, communication, finance, business, information, research, and education services). The mean reported completion of interventions was 45%. These rates are similar to adherence rates reported for digital health (50%) [62] and digital CBT (median 56%) [63] interventions and are slightly less than those reported for guided digital CBT interventions (67.5%) [64]. Mean study attrition was higher for the intervention groups (23%) than for the control groups (13%). This is in line with a review of computerized CBT [63], which reported that participants in the intervention arm were twice as likely to drop out.

### Intervention Characteristics

Over half of the studies included in this review used interventions that were predominantly based on CBT (57%). The mean duration of the interventions was 7.6 weeks, with just under half (48%) of the interventions offering some form of guidance. The mean adherence to the interventions was 45%. In a review of digital health interventions, Kelders et al [62] reported a mean duration of 10 weeks, adherence of 50%, and 76% of interventions offering some form of guidance, suggesting that occupational digital mental health interventions may differ somewhat from broader digital health interventions.

In this review, 81% of the interventions described in the studies used some form of persuasive technology: tailoring was used by 57%, self-monitoring by 43%, and tunneling by 14%. Kelders et al [62] report that for the 48 mental health studies that were included in their review of digital health interventions, tailoring was used by 90%, self-monitoring by 12%, and tunneling by 100%. The discrepancy between the number and type of persuasive technologies identified in our review and the Kelders et al [62] review is explained by differences in coding. For example, Kelders et al [62] did not code computer-mediated communication as persuasive technology, whereas we did. If a coach provided personalized feedback on assignments, we coded this as tailoring, whereas Kelders et al [62] only coded technology initiated communication (ie, when an automated message was sent). This and other differences in the coding make a comparison between the two reviews difficult.

### Meta-Analyses Findings

Our results indicate that digital mental health interventions delivered in the workplace produced a small positive effect on psychological well-being (*g*=0.37, 95% CI 0.23-0.50, k=21), and a small positive effect on work effectiveness (*g*=0.25, 95% CI 0.09-0.41, k=13).

Our findings situate occupational digital mental health interventions as comparable with other (nondigital specific) occupational interventions in terms of impact on mental health and work effectiveness. The psychological well-being effect size is smaller but not significantly different from the medium effect size reported for a meta-analysis of occupational stress management interventions (*d*=0.53 95% CI 0.36-0.69) [65] and is larger but not significantly different from the small effect sizes reported in meta-analyses of occupational resilience building programs (*d*=0.21, 95% CI 0.13-0.29) [66] and health promotion in the workplace programs (depression: *g*=0.28, 95% CI 0.12-0.44; anxiety *g*=0.29, 95% CI 0.06-0.53) [67], suggesting that on the psychological outcome, digital mental health interventions have a comparable effect with other occupational interventions. The work effectiveness effect size is comparable with the small effect size reported in a meta-analysis of work engagement interventions (*g*=0.29, 95% CI 0.12-0.46) [68], suggesting that digital mental health interventions have comparable effects with alternative approaches to enhancing engagement in the workplace.

The psychological well-being effect size for occupational digital mental health interventions in our review is also comparable with digital mental health interventions delivered in health and community settings for adults with depression [17,22] and similar to digital stress management interventions delivered in community, occupational, and health contexts [24]. Eight studies in the Heber et al [24] review also met the criteria for inclusion in the present review, but less than half of the 23 studies were set within an occupational context.

Our findings suggest that occupational digital mental health interventions are as effective at improving mental health outcomes as are other more traditional, nondigital occupational programs and other digital interventions delivered in nonoccupational settings. This is impressive given that the workplace context may impact on the way that digital mental health interventions are delivered and received. For example, it has been suggested that two of the advantages of digital health interventions compared with face-to-face or group interventions are increased accessibility, with participants being able to access at a time and a pace convenient for them [23,25,27,30,69], and increased anonymity [23,27,30]. It is these perceived advantages that researchers suggest make digital interventions particularly suited to the workplace [70]. But it is possible that these attributes don’t manifest as advantages in occupational settings; the lack of structure around “attending” digital health interventions may impact on uptake and attendance. Face-to-face or group interventions have a predetermined time for attendance during the working day, possibly with monitoring or reporting of participation to line managers. Digital mental health interventions tend to have less formal attendance with participants expected to attend at a time convenient to them. This flexibility and lack of monitoring, especially among a stressed population who may perceive themselves as time poor, may have a negative impact on intervention engagement; participants may not prioritize the time they need to engage with the intervention during their working day and may resent the intrusion of what they could perceive as work into their evening or weekends.

Furthermore, within an occupational setting, accessing digital mental health interventions may not be anonymous or even confidential. Access to the intervention may be managed through line management or occupational health; employees that do not have job autonomy may need to get permission to access the intervention during the working day, and employees working in an open plan office or sharing computer equipment may feel exposed when accessing the intervention at work. It is also possible that during the working day employees are so invested in appearing competent and strong that they are not willing or able to engage with a digital mental health intervention. The workplace may not be the appropriate setting to embrace the vulnerability that comes with acknowledging and addressing mental health challenges.

Further research is needed to gain a clearer understanding of the challenges and benefits of delivering digital mental health interventions within occupational settings. Nevertheless, despite the possibility that the workplace may provide additional challenges to the way that these interventions are delivered and received, our study has shown that occupational digital mental health interventions are effective at improving psychological well-being and work effectiveness.

### Subgroup Analyses

#### Therapeutic Approach

The results of our review would suggest that as it is currently being delivered; CBT-based occupational digital mental health interventions are not producing superior results compared with digital interventions using other psychological approaches. Subgroup analysis comparing studies in our review using approaches described by the study authors as cognitive or cognitive behavioral therapy with studies using other psychological approaches revealed that the groups are not significantly different from each other on either the psychological well-being or work effectiveness measures. These findings are contrary to the established literature.

A meta-analysis of digital psychological treatments for adult depression also found no difference between CBT and other approaches [17], but a meta-analysis of digital psychological interventions for a range of problems did report a larger effect size for interventions using CBT compared with other therapeutic approaches [71]. Furthermore, meta-analyses on digital CBT consistently report higher effect sizes than were found in this review [20,23].

One explanation for this may be that as they are currently being delivered, CBT-based digital mental health interventions are not optimized for delivery in occupational settings. In a recent review of occupational digital health, Lehr et al [16] observed that the theoretical background for many of these predominantly CBT-based interventions fails to incorporate theoretical frameworks of occupational stress. Relevant theoretical models include the effort reward imbalance model [72], the person-environment fit model (for an overview see [73]), and the job demands-control model [74]. Incorporating these frameworks into the content of occupational digital mental health interventions may make the interventions more relevant and sensitive to the workplace [16] and may increase the capacity of all psychological approaches to meet the needs of occupational groups.

#### Guidance

No significant difference was found in our review between interventions that provide guidance with those that are self-guided. This is different to the established literature, which has consistently found that guided Internet interventions are significantly superior to unguided interventions [17,22-24,28,75-78]. A review by Grist and Cavanagh [20] on computerized CBT for common mental health problems also found no significant difference in effect size between guided and unguided programs. The authors suggested caution in interpreting their findings as only 5 studies using unguided programs had been identified. Low power from a small number of studies may also be an issue for this study; consequently, we too suggest caution in interpreting these findings. Another explanation for these findings may be the failure of this review to adequately code and differentiate the extent and form of guidance that is offered to participants and the extent to which that guidance is utilized. A recent review of digital interventions for stress differentiated between (1) guided interventions, (2) adherence-focused guidance (feedback on request), and (3) unguided interventions that provided email or telephone reminders [24]. This review did not make such a distinction, differentiating solely between interventions that did not describe guidance in any form and interventions that did describe some form of guidance. Furthermore, some studies’ failure to adequately describe the in-program-support offered to participants may have resulted in some studies being wrongly categorized as unguided or guided. It is also unclear from some of the study descriptions whether support was being offered to participants outside the digital intervention, such as from an employee assistance program (EAP) or an occupational health team.

It is worth noting that the positive deviance analysis found 71% of studies in the high engagement group offered guidance compared with only 36% in the remaining studies, suggesting that there may be a link between the provision of guidance and increased engagement with occupational digital mental health interventions.

#### Targeted and Universal Populations

No significant differences were found in the review between studies that recruited a targeted population (elevated levels of depression, stress, and insomnia) and studies that targeted a universal population for either well-being or work effectiveness outcomes. However, there was a trend in both cases for studies with a targeted population to have a larger effect size, suggesting that individuals with raised levels of stress, depression, and insomnia benefit more from occupational digital mental health. One explanation for this might be that the measures used may not be sensitive to change at the lower end of the scale. Another explanation might be that participants with raised levels of psychological distress may be more motivated to implement the learning in the program and therefore produce more immediate post intervention results.

These findings are contrary to a meta-analysis on workplace resilience interventions, which found weaker effects among targeted populations compared with universal populations at post intervention [66]. That study reported that the effects of occupational resilience-building diminished sharply over time among the universal population but increased in the targeted population, suggesting that for a resilience-building program the benefits amongst a targeted population may increase with time [66].

### Positive Deviance

Maximizing engagement with, and adherence to, digital heath interventions remains a pressing concern. The partial implementation of the positive deviance approach used in this review suggests that, within an occupational setting, These findings suggest that interventions that achieve the greatest engagement and adherence offer guidance, are delivered over a shorter time frame (6 to 7 weeks), utilize secondary modalities for delivering the intervention and engaging users (ie, email and text messages), and use persuasive technology (ie, self-monitoring and tailoring). These findings echo the literature on digital health interventions. In reviews of the design features that promote adherence to digital health interventions, evidence has also been found for increased guidance [62], the shorter duration of the intervention [78], contact through email or phone [30], and incorporating tailoring and self-monitoring [79]. Meta-analyses of occupational stress management interventions [65], digital stress management in the general adult population [24], and digital psychological treatment for depression [22] also found evidence for the increased effectiveness of interventions delivered over a similar period. We would recommend the development and testing of optimized occupational digital mental health interventions based on these principles.

Only 2 of the 21 studies included in this review used a mobile phone app as their primary modality of intervention delivery. Both studies were included in the high engagement group, suggesting that app technology is a promising modality for engaging users of occupational digital mental health interventions.

### Limitations

This study highlights limitations in the broader digital mental well-being literature. One limitation is the small number of studies that measured occupational outcomes. Although the studies included in the review were aimed at employed participants and delivered within workplace contexts, most of them reported the reduction of psychological symptoms and failed to report occupational outcomes. We would recommend that future trials of psychological interventions delivered in the workplace incorporate occupational outcome measures, including work effectiveness.

Another limitation was the considerable heterogeneity that was found across the studies. This included variation in the measures used (particularly in the work effectiveness measures), variations in the guidance given and the adherence, therapeutic approach and delivery of interventions, variation in the participants including country, type of organization, role and symptom severity, and variation in the quality of the study. The large number of unclear and high-risk of bias ratings limit the quality of the studies included in the review. The variation across the studies suggests that the results of our study should be interpreted with caution. We recommend that future research uses more robust study designs.

The coding used in the review was limited by the description given about the interventions in the published literature. Many of the descriptions were short and appeared incomplete. This is a limitation described by other researchers [30,62,80]. Naturally, incomplete descriptions, especially descriptions of the persuasive technology and guidance, limit the strength of the conclusions that can be drawn here.

Other limitations specific to this review include the use in the positive deviance analysis of intervention completion and intervention group attrition as proxy measures of intervention engagement and adherence; the number of times that a participant logs in to an intervention or the number of modules that they complete cannot necessarily be taken as a measure of the extent to which they engage psychologically with the intervention [80,81]; Likewise, the extent to which participants comply with the study protocol is not a perfect measure of psychological engagement. It is reassuring to note, however, that a review of adherence and its impact on digital therapies [82] reported that module completion was found to be the adherence measure most related to outcomes in psychological health interventions. Other limitations to the review include our use of the term “psychological well-being.” We recognize that psychological well-being is more than the absence of stress or depression and that our use of the term in this review does not capture aspects of well-being such as autonomy, personal growth, functioning, and relationships with others. Finally, this review did not analyze follow-up data, so we are unable to draw conclusions on the long-term effect of digital occupational mental health programs.

### Implications

This review has demonstrated that delivering digital mental health interventions in the workplace can result in improved psychological well-being and work effectiveness. Our findings suggest that interventions that achieve the greatest engagement and adherence offer guidance, are delivered over a shorter time frame (6 to 7 weeks), utilize secondary modalities for delivering the intervention and engaging users (ie, email and text messages), and use persuasive technology (ie, self-monitoring and tailoring). Further research is needed to test these hypotheses.

We recommend that researchers and developers of occupational digital mental health interventions acknowledge the importance of the workplace setting in the content, delivery, and analysis of their interventions. We strongly recommend that therapeutic approaches incorporate relevant theoretical frameworks of occupational stress and that further research is conducted to better understand the challenges and benefits to delivering digital mental health interventions in the workplace. We also recommend that researchers incorporate in future research nonclinical measures of psychological distress and measures of occupational outcomes so that we can learn more about the psychological and occupational impact of digital mental health. A future area of research would be the long-term effect of these interventions.

### Conclusions

This review provides evidence that occupational digital mental health interventions can improve workers’ psychological well-being and increase work effectiveness and identifies intervention characteristics that may increase engagement. We recommend that researchers and intervention developers recognize that the workplace is a dynamic and complex environment that may affect the way that individuals receive and engage with digital mental health interventions.

## Appendix

Multimedia Appendix 1 Selected characteristics of included studies.

Multimedia Appendix 2 Selected characteristics of included interventions.

## Abbreviations

CBT: cognitive behavioral therapy
EAP: employee assistance program
RCT: randomized controlled trial
SD: standard deviation
WLC: wait list control
