# Supplementary material for: Improving Employee Well-Being and Effectiveness: Systematic Review and Meta-Analysis of Web-Based Psychological Interventions Delivered in the Workplace
Source: J Med Internet Res. 2017 Jul 26;19(7):e271. doi: 10.2196/jmir.7583 (PMC5550734; doi:10.2196/jmir.7583)
Supplement: Multimedia Appendix 1 [file jmir_v19i7e271_app1.pdf]

Multimedia Appendix. Selected characteristics of included studies.

Study	Population (n)	Control and data analysis	Psychological inclusion criteria	Psychological outcome (subscale)[a]	Work outcome (subscale)[a]	Study attrition at post intervention
Abbott et al, 2009 [56]	Sales managers from an Australian industrial organization (53)	Wait list control (WLC)<br><br>Intention to treat (ITT)	None	Depression, Anxiety, Stress Scale (DASS-21) (stress)	Volume of product sold (% of target met)	Intervention=53.8% (14/26)<br>Control=29.6% (8/27)
Aikens et al, 2014 [57]	Employees from a US chemical company (89)	WLC<br><br>ITT	None	Perceived Stress Scale (PSS-14)	N/A[b]	Intervention=18.2% (8/44)<br>Control=6.7% (3/45)
Allexandre et al, 2016 [52]	Employees from a US corporate call center (91[c])	WLC<br><br>ITT	None	PSS-10	Productivity	Intervention=44.4% (24/54)<br>Control=32.4% (12/37)

Study	Population (n)	Control and data analysis	Psychological inclusion criteria	Psychological outcome (subscale)[a]	Work outcome (subscale)[a]	Study attrition at post intervention
Beauchamp et al, 2005 [62]	Employed care givers of people with dementia in the United States (299)	WLC<br><br>Per protocol	None	Centre for Epidemiologic Studies-Depression scale (CES-D)	N/A	Study attrition=6.7% (22/329)
Billings et al, 2008 [54]	Employees from US technology company (309)	WLC<br><br>Per protocol	None	Symptoms of distress scale	Work Limitations Questionnaire (WLQ; output demands)	Intervention=26.6% (41/154)<br>Control=14.8% (23/155)
Birney et al, 2016 [43]	Employed adults in the United States (300)	Active control (links to websites on depression)	Mild to moderate depression (score of 10-19 on Patient Health Questionnaire	PHQ-9	Workplace outcome suite (WOS; engagement)	Intervention=6.7% (10/150)<br>Control=2.7% (4/150)

Study	Population (n)	Control and data analysis	Psychological inclusion criteria	Psychological outcome (subscale)[a]	Work outcome (subscale)[a]	Study attrition at post intervention
		ITT	[PHQ-9])			
Bolier et al, 2014 [49]	Nurses and allied professionals in a hospital in the Netherlands (366)	WLC<br><br>ITT	None	Brief symptom inventory (BSI; depression)	Utrecht Work Engagement Scale (UWES)	Intervention=54% (96/178)<br>Control=24.9% (45/188)
Cook et al, 2007 [58]	Employees for a US human resource (HR) company (419)	Active control (given print material on same subject)<br><br>Per protocol	None	Perceived stress (5-item scale)	N/A	Intervention=18.2% (38/209)<br>Control=10.9% (23/210)
Ebert et al, 2014	Teachers in	WLC	Elevated levels	Perceived	Teacher self-	Intervention=14.7%

Study	Population (n)	Control and data analysis	Psychological inclusion criteria	Psychological outcome (subscale)[a]	Work outcome (subscale)[a]	Study attrition at post intervention
[46]	Germany (150)	ITT	of stress ≥16 on CES-D	Stress Questionnaire (PSQ)	efficacy scale	(11/75) Control=8% (6/75)
Ebert et al, 2015 [47]	Teachers in Germany (128)	WLC ITT	Insomnia symptoms ≥15 on Insomnia Severity Index (ISI) and low levels of psychological detachment from work ≥15 on Cognitive Irritation Scale (CIS) subscale of	CES-D	Work related rumination (cognitive irritation sub-scale of irritation scale)	Intervention=23.4% (15/64) Control=20.3% (13/64)

Study	Population (n)	Control and data analysis	Psychological inclusion criteria	Psychological outcome (subscale)[a]	Work outcome (subscale)[a]	Study attrition at post intervention
			the IS			
Ebert et al, 2016 [44]	General working population in Germany recruited via a health insurance company (264)	WLC<br><br>ITT	Elevated levels of stress ≥22 on PSS-10	PSS-10	UWES	Intervention=9.8% (13/132)<br>Control=1.5% (2/132)
Feicht et al, 2013 [59]	Employees from two departments of a German insurance company (147)	WLC<br><br>Per protocol	None	Stress Warning Signals Scale (SWS)	N/A	Intervention=15.3% (13/85)<br>Control=8.1% (5/62)
Geraedts et al,	Employees from	Care as usual	Elevated levels	CES-D	World Health	Intervention=36.2%

Study	Population (n)	Control and data analysis	Psychological inclusion criteria	Psychological outcome (subscale)[a]	Work outcome (subscale)[a]	Study attrition at post intervention
2014 [60]	six organizations in the Netherlands: 2 banking, 2 research, 1 security, and 1 university (231)	(CAU)<br><br>ITT	of depressive symptoms≥16 on CES-D		Organization Health and Work Performance Questionnaire (WHO HPQ-4; 1 item work performance only)	(42/116)<br>Control=16.5% (19/115)
Grime 2004 [50]	National Health Service (NHS) and local authority employees recruited through	CAU<br><br>ITT	10 or more consecutive days off work for stress, anxiety or depression in last 6 months. ≥4	Hospital Anxiety and Depression Scale (HADS; Depression)	N/A	Intervention=20% (5/24)<br>Control=0% (0/24)

Study	Population (n)	Control and data analysis	Psychological inclusion criteria	Psychological outcome (subscale)[a]	Work outcome (subscale)[a]	Study attrition at post intervention
	a UK NHS occupational health department (48)		General Health Questionnaire (GHQ-12)			
Heber et al, 2016 [45]	General working population in Germany recruited via a health insurance company (264)	WLC<br><br>ITT	Elevated levels of stress ≥22 on PSS-10	PSS-10	UWES	Intervention=12.1% (16/132)<br>Control=3.8%(5/132)
Imamura et al, 2014 [55]	Employees at two Japanese information technology (IT) companies (762)	Active control (email message once a month with stress	None	Beck depression inventory (BDI)	N/A	Intervention=29.1% (111/381)<br>Control=11.8% (45/381)

Study	Population (n)	Control and data analysis	Psychological inclusion criteria	Psychological outcome (subscale)[a]	Work outcome (subscale)[a]	Study attrition at post intervention
		management info)<br><br>ITT				
Ly et al, 2014 [61]	Middle managers at medium or large employers in Sweden (73)	WLC<br><br>ITT	None	PSS-14	Multifactor Leadership Questionnaire (MLQ; transformative leadership subscales, self-report only)	Intervention=8.3% (3/36)<br>Control=(2/37) 5.4%
Phillips et al, 2014 [51]	Employees recruited through	Active control (sign posting to	≥2 on 5 of the 9 items on PHQ-9	PHQ-9	N/A	Intervention=46.2% (147/318)

Study	Population (n)	Control and data analysis	Psychological inclusion criteria	Psychological outcome (subscale)[a]	Work outcome (subscale)[a]	Study attrition at post intervention
	the occupational health sections of 3 large UK employers: transport, health, and communication (637)	websites with general info about mental health)<br><br>ITT	and employee identified that at least one item impacted on work or home life.			Control=41.1% (131/319)
Ruwaard et al, 2007 [41]	General population in the Netherlands[d] (239)	WLC<br><br>ITT	None	DASS-42 (stress)	N/A	Intervention=15.2% (27/177)<br>Control=1.6% (1/62)
Thiart et al, 2015 [48]	Teachers in Germany (128)	WLC	Clinical insomnia (≥15 on the ISI),	Penn State Worry	Work related rumination	Intervention=3.1% (2/64)

Study	Population (n)	Control and data analysis	Psychological inclusion criteria	Psychological outcome (subscale)[a]	Work outcome (subscale)[a]	Study attrition at post intervention
		ITT	experiencing work related rumination (≥15 on the CIS subscale of the irritation scale)	Questionnaire (PSWQ)	(cognitive irritation subscale of irritation scale)	Control=12.5% (8/64)
Umanodan et al, 2014 [53]	Employees from a manufacturing company in Japan (263)	WLC<br><br>ITT	None	Brief Job Stress Questionnaire (BJSQ)	Japanese version of the Utrecht Work Engagement Scale (UWES-J)	Intervention=2.8% (4/142)<br>Control=0.8% (1/121)

[a]Selected outcome used in meta-analysis.

[b]N/A: not applicable; not measured in the study.

[c]Participant number includes only the two arms of this four arm trial that have been included in this review.

[d]The study was aimed at people experiencing work related stress. Seven participants were unemployed because of workplace stress, and 10 participants suffered from stress in unpaid jobs.

References

41. Ruwaard, J., Lange, A., Bouwman, M., Broeksteeg, J., & Schrieken, B. E‐Mailed standardized cognitive behavioural treatment of work‐related stress: A randomized controlled trial. *Cognitive Behaviour Therapy* 2007; *36*(3), 179–192. PMID: 17852171

43. Birney, A. J., Gunn, R., Russell, J. K., & Ary, D. V. MoodHacker Mobile Web App With Email for Adults to Self-Manage Mild-to-Moderate Depression: Randomized Controlled Trial. *JMIR Mhealth and UHealth* 2016; *4*(1), e8. PMID: 26813737

44. Ebert, D. D., Heber, E., Berking, M., Riper, H., Cuijpers, P., Funk, B., & Lehr, D. Self-guided internet-based and mobile-based stress management for employees: results of a randomised controlled trial. *Occupational and Environmental Medicine* 2016; *73*(5), 315–323. PMID: 26884049

45. Heber, E., Lehr, D., Ebert, D. D., Berking, M., & Riper, H. Web-Based and Mobile Stress Management Intervention for Employees: A Randomized Controlled Trial. *Journal of Medical Internet Research* 2016; *18*(1), e21. PMID: 26818683

46. Ebert, D. D., Lehr, D., Boß, L., Riper, H., Cuijpers, P., Andersson, G., et al. Efficacy of an internet-based problem-solving training for teachers: results of a randomized controlled trial. *Scandinavian Journal of Work, Environment & Health*, *40*(6) 2014; PMID: 25121986

47. Ebert, D. D., Berking, M., Thiart, H., Riper, H., Laferton, J. A. C., Cuijpers, P., et al. Restoring depleted resources: Efficacy and mechanisms of change of an internet-based unguided recovery training for better sleep and psychological detachment from work. *Health Psychology* 2015; *34 Suppl*, 1240–1251. PMID: 26651465

48. Thiart, H., Lehr, D., Ebert, D. D., Berking, M., & Riper, H. Log in and breathe out: internet-based recovery training for sleepless employees with work-related strain - results of a randomized controlled trial. *Scand J Work Environ Health* 2015; 41(2): 164-74 PMID: 25590336

49. Bolier, L., Ketelaar, S. M., Nieuwenhuijsen, K., Smeets, O., Gärtner, F. R., & Sluiter, J. K. Workplace mental health promotion online to enhance well-being of nurses and allied health professionals: A cluster-randomized controlled trial. *Internet Interventions* 2014; *1*(4), 196–204. http://dx.doi.org/10.1016/j.invent.2014.10.002

50. Grime, P. R. Computerized cognitive behavioural therapy at work: a randomized controlled trial in employees with recent stress related absenteeism. *Occupational Medicine (Lond)* 2004; *54*, 353–359. PMID: 15289593

51. Phillips, R., Schneider, J., Molosankwe, I., Leese, M., Foroushani, P. S., Grime, P., et al. Randomized controlled trial of computerized cognitive behavioural therapy for depressive symptoms: Effectiveness and costs of a workplace intervention. *Psychological Medicine* 2014; *44*(4), 741–752. PMID: 23795621

52. Allexandre, D., Bernstein, A. M., Walker, E., Hunter, J., Roizen, M. F., & Morledge, T. J. A Web-Based Mindfulness Stress Management Program in a Corporate Call Center: A Randomized Clinical Trial to Evaluate the Added Benefit of Onsite Group Support. *Journal of Occupational and Environmental* Medicine 2016; 58(3), 254–264. PMID: 26949875

53. Umanodan, R., Shimazu, A., Minami, M., & Kawakami, N. Effects of computer-based stress management training on psychological well-being and work performance in Japanese employees: a cluster randomized controlled trial. *Industrial Health* 2014; *52*(6), 480–491. PMID: 25055847

54. Billings, D. W., Cook, R. F., Hendrickson, A., & Dove, D. C. A web-based approach to managing stress and mood disorders in the workforce. *Journal of Occupational and Environmental* 2008; *50*(8), 960–968. PMID: 18695455

55. Imamura, K., Kawakami, N., Furukawa, T. A., Matsuyama, Y., Shimazu, A., Umanodan, R., et al. Effects of an Internet-based cognitive behavioral therapy (iCBT) program in Manga format on improving subthreshold depressive symptoms among healthy workers: a randomized controlled trial. *PLoS ONE* 2014; *9*(5), e97167. PMID: 24844530

56. Abbott, J.-A., Klein, B., Hamilton, C., & Rosenthal, A. The Impact of Online Resilience Training for Sales Managers on Wellbeing and Work Performance. *Electronic Journal of Applied Psychology* 2009; *5*(1), 89–95.

57. Aikens, K. A., Astin, J., Pelletier, K. R., Levanovich, K., Baase, C. M., Park, Y. Y., & Bodnar, C. M. Mindfulness goes to work: Impact of an online workplace intervention. *Journal of Occupational and Environmental Medicine* 2014; *56*(7), 721–731. PMID: 24988100

58. Cook, R. F., Billings, D. W., Hersch, R. K., & Back, A. S. A field test of a web-based workplace health promotion program to improve dietary practices, reduce stress, and increase physical activity: randomized controlled trial. *Journal of Medical Internet Research* 2007; *9*(2): e17. PMID: 17581811

59. Feicht, T., Wittmann, M., Jose, G., Mock, A., Hirschhausen, von, E., & Esch, T. (2013). Evaluation of a seven-week web-based happiness training to improve psychological well-being, reduce stress, and enhance mindfulness and flourishing: a randomized controlled occupational health study. *Evidence-Based Complementary and Alternative Medicine* 2013; 676953. PMID: 24489588

60. Geraedts, A. S., Kleiboer, A. M., Wiezer, N. M., van Mechelen, W., & Cuijpers, P. Short-term effects of a web-based guided self-help intervention for employees with depressive symptoms: Randomized controlled trial. *Journal of Medical Internet Research* 2014; *16*(5), e121. PMID: 24800966

61. Ly, K. H., Asplund, K., & Andersson, G. Stress management for middle managers via an acceptance and commitment-based smartphone application: A randomized controlled trial. *Internet Interventions* 2014; 95-101 http://doi.org/10.1016/j.invent.2014.06.003

62. Beauchamp, N., Irvine, A. B., Seeley, J., & Johnson, B. Worksite based internet multimedia program for family caregivers of persons with dementia. *The Gerontologist* 2005; *45*(6), 793–801. PMID: 16326661

Source
This is a Multimedia Appendix to a full manuscript published in the J Med Internet Res, for full copyright and citation information see http://dx.doi.org/10.2196/jmir.7583
