# Supplementary material for: Improving Employee Well-Being and Effectiveness: Systematic Review and Meta-Analysis of Web-Based Psychological Interventions Delivered in the Workplace
Source: J Med Internet Res. 2017 Jul 26;19(7):e271. doi: 10.2196/jmir.7583 (PMC5550734; doi:10.2196/jmir.7583)
Supplement: Multimedia Appendix 2 [file jmir_v19i7e271_app2.pdf]

Multimedia Appendix. Selected characteristics of included interventions.

Study	Intervention description	Type[a]	Primary modality (secondary modalities)	Persuasive tech	Guidance	Length	Adherence[b]
Abbott et al, 2009 [56]	*ResilienceOnline* Enhancing 7 core skills of resilience. Delivered via video and slides.	Cognitive therapy	Website (telephone, conference call, and emails)	*Self-monitoring* using a questionnaire to measure resilience	Yes— member of staff (offer of telephone and conference call)	10 weeks	27% (7/26) completed the whole program
Aikens et al, 2014 [57]	*Mindfulness Goes to Work* Mindfulness delivered via virtual and in-person classes and Web-based training	Mindfulness	Website (email, texting, workbook, webinar, in person mtgs)	*Self- monitoring* using a weekly progress tracking survey. *Tailoring* using preprogramed emails as a	Yes— MBSR instructor	7 weeks	63.6% (28/44) completed ≥75% of the program

Study	Intervention description	Type[a]	Primary modality (secondary modalities)	Persuasive tech	Guidance	Length	Adherence[b]
				response to the survey. Opt in text messages that correspond to progress.			
Allexandre et al, 2016 [52]	*Stress Free Now* Web-based, interactive educational program based on mindfulness meditation	Mindfulness	Website (email, compact discs [CDs])	Non described	No	8 weeks	7.4% (4/54) accessed website≥4 out of 8 weeks
Beauchamp et al, 2005	*Care Giver's Friend: Dealing with*	Stress and coping model	Website (email)	*Tailoring* via a questionnaire that	No	4.3 weeks	10.6% (16/150)

Study	Intervention description	Type[a]	Primary modality (secondary modalities)	Persuasive tech	Guidance	Length	Adherence[b]
[62]	*Dementia* Multimedia intervention with text material and videos that model positive caregiving strategies			produces links tailored to the individual			visited≥4 times (19% (29/150) visited ≥2)
Billings et al, 2008 [54]	*Stress and Mood Management* Modular, multi media program. Audio narrated with video and graphics.	Cognitive behavioral therapy (CBT)	Website	Tailoring through an embedded assessment instrument	No	13 weeks	3.2% (5/154) accessed stress management module≥4 times
Birney et al,	*MoodHacker.*	CBT and	Mobile	*Tunneling* through	No	6	On average

Study	Intervention description	Type[a]	Primary modality (secondary modalities)	Persuasive tech	Guidance	Length	Adherence[b]
2016 [43]	Sequenced content delivered through in-app messaging, articles, and videos.	positive psychology	phone app (emails)	sequenced content. *Self-monitoring* of mood and activities		weeks	participants logged in 16 times (standard deviation [SD] 13.3)
Bolier et al, 2014 [49]	Based on personal screening, participants offered access to a number of Web-based interventions	CBT	Website (email)	*Tailoring* through automated personalized feedback and signposting to interventions	No	13 weeks	5.1% (9/178) started≥1 module
Cook et al, 2007 [58]	*Health connection.* Multimedia, health	Social cognitive	Website	Non described	No	13 weeks	15.4% (32/209)

Study	Intervention description	Type[a]	Primary modality (secondary modalities)	Persuasive tech	Guidance	Length	Adherence[b]
	promotion for the workplace designed to improve dietary practices, reduce stress, and increase physical activity	theory					accessed the stress management module >2
Ebert et al, 2015 [47]	*GET.ON Recovery* 6 sessions including articles, exercises, testimonies, audio, and video	Cognitive-behavioral and metacognitive techniques for insomnia	Website	*Tailoring* through content being modified depending on response options. Self- monitoring through recovery diary	No	6 weeks	48.4% (31/64) completed all sessions

Study	Intervention description	Type[a]	Primary modality (secondary modalities)	Persuasive tech	Guidance	Length	Adherence[b]
Ebert et al, 2016 [44]	*GET.ON Stress* 7 sessions (plus optional booster session and 8 optional modules) including text, exercises, testimonials, audio, and video	Transactional model of stress. Problem solving and emotional regulation.	Website (text messages)	*Tailoring* through optional modules and content being modified depending on response option. Self-monitoring through stress diary	No	7 weeks	42% (55/131) completed session 7/7
Feicht et al, 2013 [59]	Happiness training focusing on exercises to achieve a positive psychological state	Positive psychology	Website (emails)	Non described	No	7 weeks	N/A[g]

Study	Intervention description	Type[a]	Primary modality (secondary modalities)	Persuasive tech	Guidance	Length	Adherence[b]
Geraedts et al, 2014 [60]	*Happy@Work.* 6 weekly sessions including information, examples, and assignments. Weekly assignments submitted to a coach	Cognitive therapy and problem solving	Website (emails)	*Tunneling* participants unable to start new session until they receive feedback on previous session. *Tailoring* through individualized feedback from a coach	Yes—coach (feedback on assignments)	6 weeks	26.7% (32/116) completed 6/6 lessons
Grime 2004 [50]	*Beating the Blues.* Interactive CBT program	CBT	Standalone computer	*Tunneling*, you complete one module before	No	8 weeks	66.7% (16/24) completed 8/8 sessions

Study	Intervention description	Type[a]	Primary modality (secondary modalities)	Persuasive tech	Guidance	Length	Adherence[b]
				starting another<br>Self-monitoring through weekly progress reports			
Heber et al, 2016 [45]	*GET.ON Stress*<br>Seven sessions (plus optional booster session) including psycho-education, interactive exercises	Transactional model of stress	Website (emails, text messages)	*Tailoring* through personalized feedback	Yes—coach (feedback on sessions)	7 weeks	70.5% (93/132) completed 7/7 sessions
Imamura et al, 2014 [55]	*Internet CBT Program: Useful*	CBT	Website (emails)	*Tailoring* through personalized	Yes—clinical psychologist	6 weeks	64.8% (247/381)

Study	Intervention description	Type[a]	Primary modality (secondary modalities)	Persuasive tech	Guidance	Length	Adherence[b]
	mental health solutions series for business Internet CBT using Manga (Japanese comic) story delivered over 6 lessons			feedback	(feedback on assignments)		completed 6/6 sessions
Ly et al, 2014 [61]	Mobile phone stress management intervention, 6 modules, each module has a short audio lecture, plus information and	Acceptance and commitment therapy (ACT)	Mobile phone app (text messages)	*Self-monitoring* through personal statistics on the app	Yes—therapist (one way text messages)	6 weeks	44.4% (16/36) adhered to intervention for 6/6 weeks

Study	Intervention description	Type[a]	Primary modality (secondary modalities)	Persuasive tech	Guidance	Length	Adherence[b]
	exercises						
Phillips et al, 2014 [51]	*MoodGYM* Interactive Web program, 5 modules, includes exercises, assessments, audio, and a game[i]	CBT	Website	Non described	No (phone calls as part of research only)	5 weeks	28.2% (90/318) considered high users (≥50% of intervention)
Ruwaard et al, 2007 [41]	7 weekly modules delivered via email. Feedback given on homework	CBT	Email	*Tailoring* of emails sent to participants	Yes—therapist (feedback on assignments)	7 weeks	72.3% (128/177) received treatment (the extent of this is not defined)

Study	Intervention description	Type[a]	Primary modality (secondary modalities)	Persuasive tech	Guidance	Length	Adherence[b]
Thiart et al, 2015 [48]	*GET.ON Recovery* 6 modules with homework. Supported by coaches via email messaging, weekly feedback on exercises	Cognitive behavioral therapy for insomnia (CBT-I)	Website (emails)	Tailoring through feedback on homework *Self-monitoring* through a recovery diary	Yes—coach (feedback on exercises and answering questions)	6 weeks	95.3% (61/64) completed 6/6 modules
Umanodan et al, 2014 [53]	Computer-based, self-paced stress management training incorporating	Cognitive behavioral techniques	Website (emails)	*Self-monitoring* of skills	Yes—coordinator (emails sent via the coordinator)	7 weeks	89.4% (127/142) competed all content

Study	Intervention description	Type[a]	Primary modality (secondary modalities)	Persuasive tech	Guidance	Length	Adherence[b]
	behavioral, communication and cognitive techniques						

[a]As described by the authors.

[b]Highest level of intervention completion reported by the authors.

[c]Description taken from website moodgym.anu.edu.au.

References

41. Ruwaard, J., Lange, A., Bouwman, M., Broeksteeg, J., & Schrieken, B. E-Mailed standardized cognitive behavioural treatment of work-related stress: A randomized controlled trial. *Cognitive Behaviour Therapy* 2007; *36*(3), 179–192. PMID: 17852171

43. Birney, A. J., Gunn, R., Russell, J. K., & Ary, D. V. MoodHacker Mobile Web App With Email for Adults to Self-Manage Mild-to-Moderate Depression: Randomized Controlled Trial. *JMIR Mhealth and UHealth* 2016; *4*(1), e8. PMID: 26813737

44. Ebert, D. D., Heber, E., Berking, M., Riper, H., Cuijpers, P., Funk, B., & Lehr, D. Self-guided internet-based and mobile-based stress management for employees: results of a randomised controlled trial. *Occupational and Environmental Medicine* 2016; *73*(5), 315–323. PMID: 26884049

45. Heber, E., Lehr, D., Ebert, D. D., Berking, M., & Riper, H. Web-Based and Mobile Stress Management Intervention for Employees: A Randomized Controlled Trial. *Journal of Medical Internet Research* 2016; *18*(1), e21. PMID: 26818683

46. Ebert, D. D., Lehr, D., Boß, L., Riper, H., Cuijpers, P., Andersson, G., et al. Efficacy of an internet-based problem-solving training for teachers: results of a randomized controlled trial. *Scandinavian Journal of Work, Environment & Health*, *40*(6) 2014; PMID: 25121986

47. Ebert, D. D., Berking, M., Thiart, H., Riper, H., Laferton, J. A. C., Cuijpers, P., et al. Restoring depleted resources: Efficacy and mechanisms of change of an internet-based unguided recovery training for better sleep and psychological detachment from work. *Health Psychology* 2015; *34 Suppl*, 1240–1251. PMID: 26651465

48. Thiart, H., Lehr, D., Ebert, D. D., Berking, M., & Riper, H. Log in and breathe out: internet-based recovery training for sleepless employees with work-related strain - results of a randomized controlled trial. *Scand J Work Environ Health* 2015; 41(2): 164-74 PMID: 25590336

49. Bolier, L., Ketelaar, S. M., Nieuwenhuijsen, K., Smeets, O., Gärtner, F. R., & Sluiter, J. K. Workplace mental health promotion online to enhance well-being of nurses and allied health professionals: A cluster-randomized controlled trial. *Internet Interventions* 2014; *1*(4), 196–204. http://dx.doi.org/10.1016/j.invent.2014.10.002

50. Grime, P. R. Computerized cognitive behavioural therapy at work: a randomized controlled trial in employees with recent stress related absenteeism. *Occupational Medicine (Lond)* 2004; *54*, 353–359. PMID: 15289593

51. Phillips, R., Schneider, J., Molosankwe, I., Leese, M., Foroushani, P. S., Grime, P., et al. Randomized controlled trial of computerized cognitive behavioural therapy for depressive symptoms: Effectiveness and costs of a workplace intervention. *Psychological Medicine* 2014; *44*(4), 741–752. PMID: 23795621

52. Allexandre, D., Bernstein, A. M., Walker, E., Hunter, J., Roizen, M. F., & Morledge, T. J. A Web-Based Mindfulness Stress Management Program in a Corporate Call Center: A Randomized Clinical Trial to Evaluate the Added Benefit of Onsite Group Support. *Journal of Occupational and Environmental* Medicine 2016; 58(3), 254–264. PMID: 26949875

53. Umanodan, R., Shimazu, A., Minami, M., & Kawakami, N. Effects of computer-based stress management training on psychological well-being and work performance in Japanese employees: a cluster randomized controlled trial. *Industrial Health* 2014; *52*(6), 480–491. PMID: 25055847

54. Billings, D. W., Cook, R. F., Hendrickson, A., & Dove, D. C. A web-based approach to managing stress and mood disorders in the workforce. *Journal of Occupational and Environmental* 2008; *50*(8), 960–968. PMID: 18695455

55. Imamura, K., Kawakami, N., Furukawa, T. A., Matsuyama, Y., Shimazu, A., Umanodan, R., et al. Effects of an Internet-based cognitive behavioral therapy (iCBT) program in Manga format on improving subthreshold depressive symptoms among healthy workers: a randomized controlled trial. *PLoS ONE* 2014; *9*(5), e97167. PMID: 24844530

56. Abbott, J.-A., Klein, B., Hamilton, C., & Rosenthal, A. The Impact of Online Resilience Training for Sales Managers on Wellbeing and Work Performance. *Electronic Journal of Applied Psychology* 2009; *5*(1), 89–95.

57. Aikens, K. A., Astin, J., Pelletier, K. R., Levanovich, K., Baase, C. M., Park, Y. Y., & Bodnar, C. M. Mindfulness goes to work: Impact of an online workplace intervention. *Journal of Occupational and Environmental Medicine* 2014; *56*(7), 721–731. PMID: 24988100

58. Cook, R. F., Billings, D. W., Hersch, R. K., & Back, A. S. A field test of a web-based workplace health promotion program to improve dietary practices, reduce stress, and increase physical activity: randomized controlled trial. *Journal of Medical Internet Research* 2007; *9*(2): e17. PMID: 17581811

59. Feicht, T., Wittmann, M., Jose, G., Mock, A., Hirschhausen, von, E., & Esch, T. (2013). Evaluation of a seven-week web-based happiness training to improve psychological well-being, reduce stress, and enhance mindfulness and flourishing: a randomized controlled occupational health study. *Evidence-Based Complementary and Alternative Medicine* 2013; 676953. PMID: 24489588

60. Geraedts, A. S., Kleiboer, A. M., Wiezer, N. M., van Mechelen, W., & Cuijpers, P. Short-term effects of a web-based guided self-help intervention for employees with depressive symptoms: Randomized controlled trial. *Journal of Medical Internet Research* 2014; *16*(5), e121. PMID: 24800966

61. Ly, K. H., Asplund, K., & Andersson, G. Stress management for middle managers via an acceptance and commitment-based smartphone application: A randomized controlled trial. *Internet Interventions* 2014; 95-101 http://doi.org/10.1016/j.invent.2014.06.003

62. Beauchamp, N., Irvine, A. B., Seeley, J., & Johnson, B. Worksite based internet multimedia program for family caregivers of persons with dementia. *The Gerontologist* 2005; *45*(6), 793–801. PMID: 16326661

Source
This is a Multimedia Appendix to a full manuscript published in the J Med Internet Res, for full copyright and citation information see http://dx.doi.org/10.2196/jmir.7583
